# An association analysis of lipid profile and diabetic cardiovascular autonomic neuropathy in a Chinese sample

**DOI:** 10.1186/s12944-016-0287-3

**Published:** 2016-07-26

**Authors:** Lige Song, Linuo Zhou, Zihui Tang

**Affiliations:** 1 Department of Endocrinology and Metabolism, Shanghai Tongji Hospital, Tongji University School of Medicine, Shanghai, 200065 China; 2 Department of Endocrinology and Metabolism, Fudan University Huashan Hospital, Shanghai, China

**Keywords:** Diabetic cardiac autonomic neuropathy, Association, Lipid profile, Chinese sample

## Abstract

**Background:**

Recent studies have shown that triglyceride (TG), low-density lipoprotein cholesterol (LDL), and high-density lipoprotein cholesterol (HDL) are related to the prevalence of cardiovascular autonomic neuropathy (CAN). However, little is known about the association of lipid profile with diabetic cardiovascular autonomic neuropathy (DCAN), or its severity in the Chinese population. The purpose of this study is to explore the extent of this phenomenon using a Chinese sample.

**Methods:**

A subgroup analysis on 455 diabetic patients with undiagnosed DCAN was performed to evaluate the relationships of lipids profile and DCAN. DCAN was diagnosed if there were at least two abnormal cardiovascular autonomic reflex test results, based on short-term heart rate variability tests. Multivariable logistic regression (MLR)was carried out to control potential confounders for determining the independent association of variables with DCAN in different models.

**Results:**

MLR analysis indicated that TG was significantly and independently associated with DCAN when controlling for confounding factors (*P <* 0.1 for two models). Additionally, TG combined with TC (LRS-1) and LDL (LRS-2) was associated with this outcome (*P <* 0.1 for LRS-1 and LRS-2).

**Conclusion:**

Our findings indicate that TG and the severity of lipids profile is significantly and independently associated with DCAN, respectively.

**Trial registration:**

ClinicalTrials.gov Identifier: NCT02461472, retrospectively registered 2 Jun, 2015

## Background

Dyslipidemia has become one of the most prevalent non-communicable diseases worldwide, as reported by the Global Burden of Disease (GBD) studies [1, 2]. Its symptoms include elevated levels of total cholesterol (TC), low-density lipoprotein cholesterol (LDL), triglycerides (TG), or decreased high-density lipoprotein cholesterol (HDL). Following lifestyle changes in China during the past 30 years, the rate of dyslipidemia in China was 18.6 % in 2004, or about 160 million people. Despite the huge number affected, only 3.5 % of men and 3.4 % and women were treated, with 1.9 % and 1.5 % being controlled, respectively [3]. Recent data has shown that only 55.9 % of outpatients in the top-ranked hospitals in the major cities of China were treated for dyslipidemia, with only 39.4 % of them achieving the LDL goal [4].

Dyslipidemia is considered an important modifiable risk factor for cardiometabolic (CM) diseases, and causes morbidity and mortality worldwide [5]. Data published recently indicates that CM risk factors are highly prevalent among youths and young adults, which poses a high risk of death from diabetes and cardiovascular disease in China [6]. The Chinese healthcare system is heavily burdened by CM diseases, which are now the leading causes of morbidity and mortality in China [7, 8]. Dyslipidemia was highly common in type 2 diabetes (T2DM) patients, and was up to 67.1 % in 2015 [4]. Dyslipidemia in T2DM is characterized by increased TG and LDL decreased HDL, and increased free fatty acid. Patients with both diabetes and dyslipidemia usually have a higher risk of cardiovascular diseases, and experience more severe clinical outcomes when compared to nondiabetic patients [9, 10].

Recent data has demonstrated that the TG/HDL ratio and a smaller mean LDL particle size were related to the prevalence of cardiac autonomic neuropathy in women with T2DM in Korea [11]. However, little is known about the association of lipid profile with DCAN, or its severity in the Chinese population. Previously, we performed a study on a Chinese sample to analyze this, and to explore the relationship between blood pressure profiles and DCAN, which revealed that systolic blood pressure and severity of blood pressure profiles was significantly and independently associated with DCAN [12]. In this study, we focused on exploring the extent to which association of lipid profiles and its severity with DCAN in a Chinese sample.

## Methods

### Study population

As mentioned earlier [13], we carried out a risk factors survey for CAN on a random sample of the Chinese population. Participants were recruited from rural and urban communities in Shanghai. As described earlier [12], a subgroup analysis was performed on diabetic participants with undiagnosed CAN aged 30–80 years. We analyzed subjects in screening visits between 2011 and 2013. Some subjects were excluded from the study to eliminate potential confounding factors that may have influenced their CA function. Briefly, the exclusion criteria were as follows: history or findings of arrhythmia and hyperthyroidism or hypothyroidism; pregnancy or lactation; and/or serious hepatic or renal dysfunctions (GFR < 30 mL/min/1.73 m^2^). A total of 455 diabetic participants with complete clinical baseline data were available for the DCAN risk factor analysis. Written consent was obtained from all patients before the study.

### Ethics statement

This study was reviewed and received ethical approval from the Ethics Committee at the Shanghai Tongji Hospital. Permission to conduct the study was granted by the Shanghai Tongji Hospital. The methods were carried out in accordance with the approved guidelines. Written informed consent was obtained from all study participants.

### Measurement

As mentioned earlier [12], the participants were interviewed for the documentation of medical histories, medication, and history of smoking habits. Laboratory assessments of cardiovascular disease risk factors were completed, along with standardized examination for heart rate variability (HRV). All subjects underwent a complete clinical baseline characteristics evaluation after an eight-hour fasting, which included history and physical examination, heart rate and blood pressure, fasting serum glucose and insulin, and fasting plasma lipids. The assessments of demographical information, lipids profiles, glucose profiles, renal function, indices of HRV, and medical history were detailed earlier [12]. The day-to-day and inter-assay coefficients of variation at the central laboratory in our hospital, for all analyses, were between 1 % and 3 %. Definitions of HTN, body mass index (BMI), DM, and MetS were detailed in our earlier studies [12, 13].

### The study outcome

As mentioned in our earlier studies, we used short-term HRV to evaluate CA function. HRV was measured non-invasively by power spectral analysis. Before the CA function assessment, participants were asked to avoid alcohol, smoking, and coffee for 24 h to help bring about a calm and quiet condition. Subjects were studied while awake in the supine position after 20 min of rest. Testing times were between 8:00 and 11:00 in the morning. A type-I FDP-1 HRV non-invasive detecting system was used with software version 2.0 (Department of Biomedical Engineering of Fudan University, Shanghai, China). In this study, CAN was diagnosed from the results of at least two abnormal cardiovascular autonomic reflex test results that were based on short-term HRV tests [14–16].

### Statistical analysis

Continuous variables were detected following normal distribution, using the Kolmogorov-Smirnov Test. Variables that were not normally distributed were log-transformed to approximate normal distribution for analysis. Results are described as mean ± SD or median, unless stated otherwise. Differences in variables between male and female participants were determined by unpaired *t*-test. Between groups, differences in properties were assessed by *χ*^*2*^ analysis.

We performed difference analyses on the prevalence of DCAN among lipid profile indices with category variables. According to clinical reference values, TC was categorized by trinary variables (code 0: <5.18 mmol/L, code 1: 5.18–6.19 mmol/L, and code 2: > 6.19 mmol/L),TG was categorized by trinary variables (code 0: <1.71 mmol/L, code 1: 1.71–2.25 mmol/L, and code 2: > 2.25 mmol/L), LDL was categorized by trinary variables(code 0: <3.37 mmol/L, code 1: 3.37–4.14 mmol/L, and code 2: > 4.14 mmol/L), and HDL was categorized by binary variables (code 0: ≥1.04 mmol/L and code 1: <1.04 mmol/L). Next, groups with similar prevalence of DCAN were combined into one group so as to gain lipid profile indices categorized by binary variables.

Univariate logistic regression, to include lipid profiles with continuous variables, was performed to determine variables associated with DCAN and to estimate confounding factors that could possibly disturb the relation between lipid profiles and DCAN.A lipid profile risk score (LRS) was calculated for associations between severity of lipid profile and DCAN. In this study, LRS-1 was the sum of TC and TG with binary variables. Similarly, LRS-2, LRS-3, and LRS-4 were derived from TG and LDL, TG and HDL, and HDL and LDL, respectively. Multivariable logistic regression (MLR) was carried out to control potential confounders for determining the independent association of variables with DCAN in six models. The results were analyzed using the Statistical Package for Social Sciences for Windows, version 16.0 (SPSS, Chicago, IL, USA). Tests were two-sided, and a p-value of < 0.05 was considered significant. For multiple variable analysis, a *p*-value of <0.10 was also considered significant.

## Results

### Clinical characteristics of participants

The baseline clinical characteristics of the 455 diabetic participants were detailed earlier [12], and are also listed in Table 1. There were 208 males and 247 females (mean age62.17 ± 8.37 years) in the complete sample. There were significant differences in TC, LDL, and HDL levels between males and females (P value <0.05), but none in TG between the two groups (P value =0.961). The level of FPG was higher in males compared to females (P value =0.006). Significant differences in the parameters of renal function between males and females were reported (P value <0.001). The mean duration of DM and HTN was 5.24 and 6.42 years in the entire sample. The prevalence of HTN, MetS, and DCAN was 63.96 %, 72.53 %, and 29.01 % in the whole sample, respectively.

**Table 1:** The clinical baseline characteristics of individuals

Variable	Female	Male	Total sample	*P* value
Demographic information				
N	247	208	455	-
Age year	62.17 ± 8.37	63.54 ± 8.84	62.8 ± 8.61	0.016
Height cm	157.2 ± 5.99	167.95 ± 6.33	162.12 ± 8.15	<0.001
Weight kg	62.9 ± 11.3	71.05 ± 10.45	66.63 ± 11.65	<0.001
SBP mmHg	134.95 ± 21.33	133.55 ± 19.02	134.3 ± 20.3	0.305
DBP mmHg	81.2 ± 10.1	80.93 ± 10.16	81.08 ± 10.12	0.690
Lipid profile				
TCmmol/L	5.64 ± 1.08	5.06 ± 1.07	5.38 ± 1.11	<0.001
TG mmol/L	1.99 ± 1.03	1.99 ± 1.34	1.99 ± 1.18	0.961
HDL mmol/L	1.38 ± 0.29	1.19 ± 0.28	1.3 ± 0.31	<0.001
LDL mmol/L	3.4 ± 0.86	3.14 ± 0.82	3.28 ± 0.85	<0.001
Glucose profile				
FPG mmol/L	7.11 ± 2.56	7.61 ± 2.82	7.34 ± 2.69	0.006
PBG mmol/L	11.9 ± 4.25	12.07 ± 4.62	11.98 ± 4.42	0.583
FINSuIml	11.09 ± 24.53	9.68 ± 24.23	10.45 ± 24.39	0.388
Renal function				
SCr μmolL	73.35 ± 23.1	90.93 ± 21.54	81.37 ± 24.04	<0.001
UA μmolL	280.17 ± 77.45	319.48 ± 89.66	298.09 ± 85.47	<0.001
HRV indices				
HR bpm	74.96 ± 9.63	75.29 ± 11.27	75.11 ± 10.41	0.634
TP ms^2^	763.34 ± 635.42	728.25 ± 734.89	747.3 ± 682.53	0.440
LF ms^2^	152.57 ± 145.95	183.19 ± 293.24	166.57 ± 225.93	0.042
HF ms^2^	163.58 ± 193.2	138.57 ± 182.1	152.15 ± 188.51	0.046
LF/HF	1.66 ± 1.91	2.05 ± 2.33	1.84 ± 2.12	0.006
Medical history				
Smokingyes,%	4(1.62 %)	85(40.87 %)	89(19.56 %)	<0.001
DMD year	4.86 ± 6.29	5.73 ± 6.62	5.24 ± 6.45	0.063
HTNyes,%	318(64.37 %)	264(63.46 %)	582(63.96 %)	0.776
HTND year	5.62 ± 9.05	7.41 ± 10.96	6.42 ± 9.99	0.008
MetSyes,%	187(75.71 %)	143(68.75 %)	330(72.53 %)	0.019
DCANyes,%	67(27.13 %)	65(31.25 %)	132(29.01 %)	0.172

Note: *SBP* systolic blood pressure, *DBP* diastolic blood pressure, *FPG* fasting plasma glucose, *PBG* plasma blood glucose, *FINS* fasting blood insulin, *TC* serum total cholesterol, *TG* triglyceride, *HDL* high-density lipoprotein cholesterol, *LDL* low density lipoprotein cholesterol, *SCr* serum creatinine, *HR* heart rate, *TP* total power of variance, *LF* low frequency, *HF* high frequency, *MetS* metabolic syndrome, *DMD* diabetes duration, *HTN* Hypertension, *HTND* Hypertension duration, *DCAN* diabetic cardiovascular autonomic neuropathy

### Difference analysis in DCAN prevalence among lipid profile

**Fig. 1:**
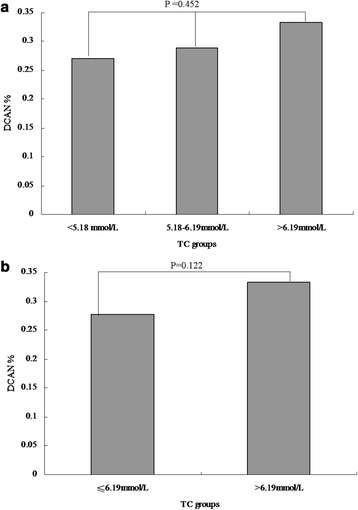
Comparison of prevalence of diabetic cardiovascular autonomic neuropathy (DCAN) according to serum total cholesterol (TC). **a**: Comparison of DCAN prevalence according to TC with trinary variables. DCAN prevalence was 27.01 %, 28.87 % and 33.33 % in the three groups, respectively. No significant differences among the three groups were reported (*P* = 0.452). **b**: Comparison of DCAN prevalence according to TC with binary variables. DCAN prevalence was 27.76 % and 33.33 % in the two groups, respectively. No significant differences between the two groups were reported (*P* = 0.122)

**Fig. 2:**
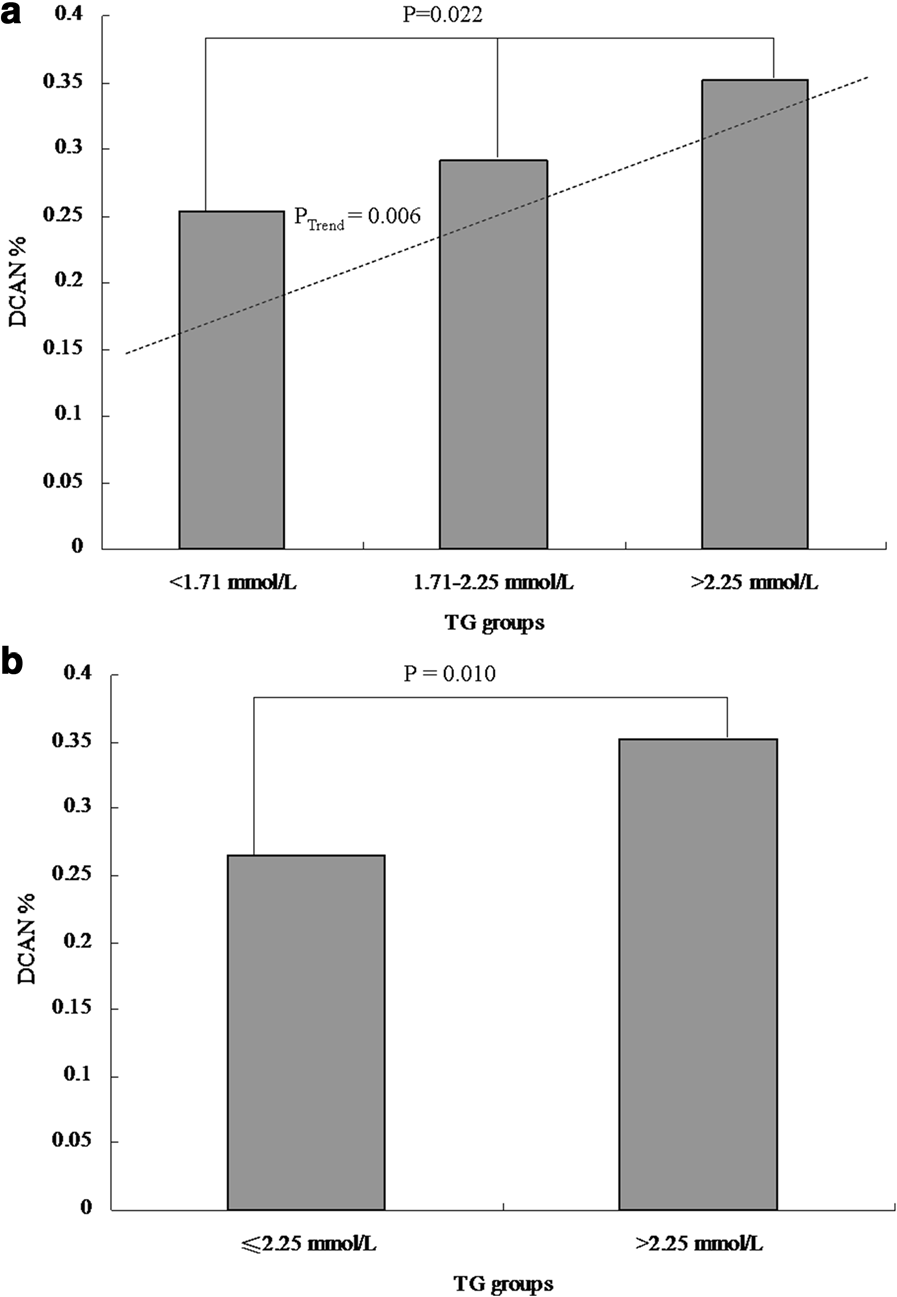
Comparison of prevalence of diabetic cardiovascular autonomic neuropathy (DCAN) according to triglyceride (TG). **a**: Comparison of DCAN prevalence according to TG with trinary variables. DCAN prevalence was 25.34 %, 29.13 % and 35.25 % in the three groups, respectively. A significant differences among the three groups were reported (*P* =0.022 and *P* for trend = 0.006). **b**: Comparison of DCAN prevalence according to TG with binary variables. DCAN prevalence was 26.54 % and 35.25 % in the two groups, respectively. A significant differences between the two groups were reported (*P* = 0.010)

**Fig. 3:**
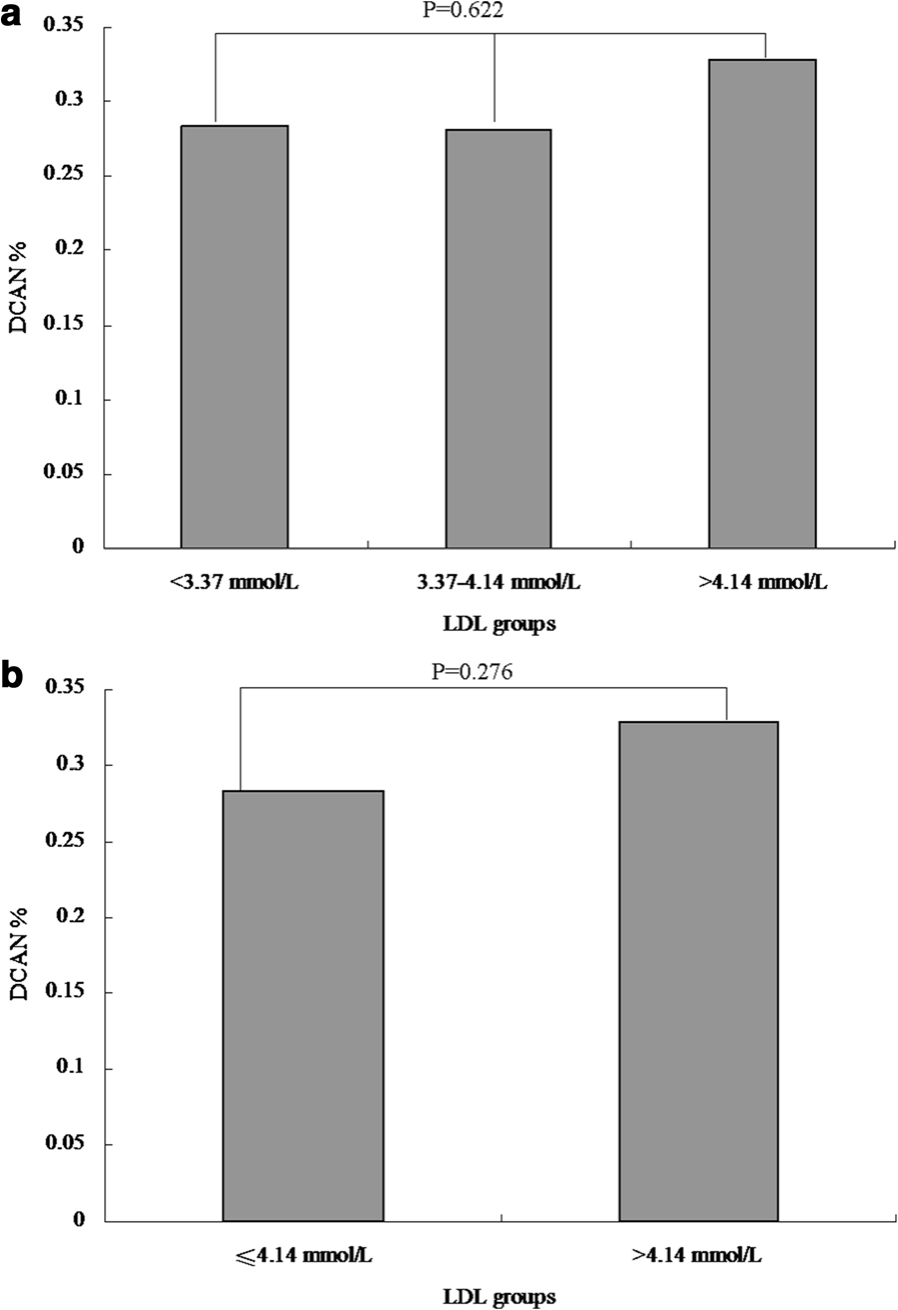
Comparison of prevalence of diabetic cardiovascular autonomic neuropathy (DCAN) according tolow density lipoprotein cholesterol (LDL). **a**: Comparison of DCAN prevalence according to LDL with trinary variables. DCAN prevalence was 28.40 %,28.13 % and 32.86 % in the three groups, respectively. No significant differences among the three groups were reported (*P* =0.622). **b**: Comparison of DCAN prevalence according to LDL with binary variables. DCAN prevalence was 28.31 % and 32.86 % in the two groups, respectively. A significant differences between the two groups were reported (*P* = 0.276)

**Fig. 4:**
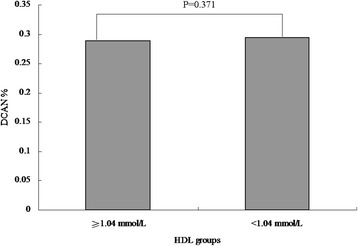
Comparison of prevalence of diabetic cardiovascular autonomic neuropathy (DCAN) according tohigh-density lipoprotein cholesterol (HDL). Comparison of DCAN prevalence according to HDL. DCAN prevalence was 28.89%n and 29.47 % in the two groups, respectively. There were no significant differences between the two groups (*P* = 0.371)

There were no significant differences in DCAN prevalence among the three TC groups (27.01 % vs. 28.87 % vs. 33.33 %, *P* = 0.452, Fig. 1a). Similarly, no significant differences between the two groups were reported (27.76 % vs. 33.33 %, *P* = 0.122, Fig. 1b). The DCAN prevalence was 25.34 %, 29.13 %, and 35.25 % in the three TG groups, respectively. However, a significant difference between the two groups was reported (*P* =0.022 and *P* for trend = 0.006, Fig. 2a). Additionally, the DCAN prevalence was significantly higher in diabetic participants with a high TG level, as compared to those with a low TG level (26.54 % vs. 35.25 %, *P* = 0.010, Fig. 2b). In the LDL groups, no significant differences among the three groups were reported (28.40 % vs. 28.13 % vs. 32.86 %, *P* =0.622, Fig. 3a). There were also no significant differences between the two groups (28.31 % vs. 32.86 %, *P* = 0.276, Fig. 3b). Similarly, there were no significant differences between the high HDL and low HDL groups (28.89 % vs. 29.47 %, *P* = 0.371, Fig. 4).

### Association analysis between lipid profile and DCAN

To estimate the association of various clinical factors and DCAN univariate logistic regression models were developed to include lipids profiles, age, gender, BMI, glucose profiles, renal functions, and medical history. As described earlier [12], the univariate logistic analyses indicated that TG, age, BMI, glucose profiles, HTND, DMD, and MetS were significantly associated with DCAN (*P <* 0.05 for all, Table 2); however, there were no significant associations of TC, LDL, and HDL with DCAN (*P >*0.05 for all).

**Table 2:** Univariate analysis to include independent variables for diabetic cardiovascular autonomic neuropathy

Variable	*β*	SE	*P* value	OR	95 % CI
Lipid profile					
TC mmol/L	0.067	0.065	0.308	1.069	0.94–1.215
TG mmol/L	0.257	0.06	<0.001	1.293	1.15–1.455
HDL mmol/L	−0.231	0.243	0.340	0.793	0.493–1.277
LDL mmol/L	−0.040	0.086	0.645	0.961	0.811–1.138
Covariance					
Age years	0.035	0.009	<0.001	1.036	1.018–1.054
Gender male	0.200	0.146	0.172	1.221	0.917–1.627
BMI kg/cm^2^	0.029	0.012	0.043	1.03	1.01–1.07
SBP mmHg	0.004	0.004	0.242	1.004	0.997–1.011
DBP mmHg	0.002	0.007	0.806	1.002	0.988–1.016
FPG mmol/L	0.098	0.026	<0.001	1.103	1.048–1.161
PBG mmol/L	0.081	0.017	<0.001	1.084	1.049–1.121
FINS uml	0.006	0.003	0.031	1.006	1.001–1.012
SCr μmolL	0.005	0.003	0.073	1.005	1.00–1.011
HR bpm	0.091	0.009	<0.001	1.095	1.076–1.114
Smoking yes	0.210	0.18	0.242	1.234	0.868–1.756
DMD years	0.031	0.012	0.010	1.031	1.007–1.056
HTN yes	0.120	0.153	0.433	1.128	0.835–1.523
HTND years	0.014	0.007	0.050	1.014	1.00–1.028
MetS yes	0.527	0.175	0.003	1.694	1.202–2.387

Note: *SBP* systolic blood pressure, *DBP* diastolic blood pressure, *FPG* fasting plasma glucose, *PBG* plasma blood glucose, *FINS* fasting blood insulin, *TC* serum total cholesterol, *TG* triglyceride, *HDL* high-density lipoprotein cholesterol, *LDL* low density lipoprotein cholesterol, *SCr* serum creatinine, *HR* heart rate, *MetS* metabolic syndrome, *HTN* Hypertension

Multiple variables logistic regression to include lipids profile, and controlling for potential confounding factors of age, gender, smoking, BMI, blood pressure, glucose profile, renal function, medical history, indicated that there was a significant association between TG and DCAN (P value =0.036OR = 1.25, 95 % CI: 1.015–1.54 for model 1, and P value = 0.096OR = 1.427, 95 % CI: 0.939–2.167 for model 2, Table 3). However, there was no significant association of TC, LDL, or HDL with DCAN (*P <* 0.05 for all).

**Table 3:** Association analysis between lipid profile for diabetic cardiovascular autonomic neuropathy using multiple variable logistic regression analysis

Model	Variable	β	SE	*P* value	OR	95 % CI
Model 1						
	TC	0.273	0.265	0.304	1.313	0.781–2.208
	TG	0.223	0.106	0.036	1.25	1.015–1.54
	LDL	−0.454	0.304	0.136	0.635	0.35–1.154
	HDL	0.545	0.380	0.151	1.725	0.819–3.631
Model 2						
	TC	0.130	0.322	0.686	1.139	0.606–2.139
	TG	0.355	0.213	0.096	1.427	0.939–2.167
	LDL	0.031	0.337	0.928	1.031	0.533–1.996
	HDL	0.358	0.250	0.152	1.430	0.876–2.336

Note: Model 1: lipid profiles with continuous variable, Model 2: lipid profiles with binary variable

*TC* serum total cholesterol, *TG* triglyceride, *LDL* low density lipoprotein cholesterol, *HDL* high-density lipoprotein cholesterol, *BMI* body mass index; all models adjusted for age, gender, smoking, BMI, blood pressure, glucose profile, renal function, medical history

### Association analysis between severity of LRS and DCAN

**Fig. 5:**
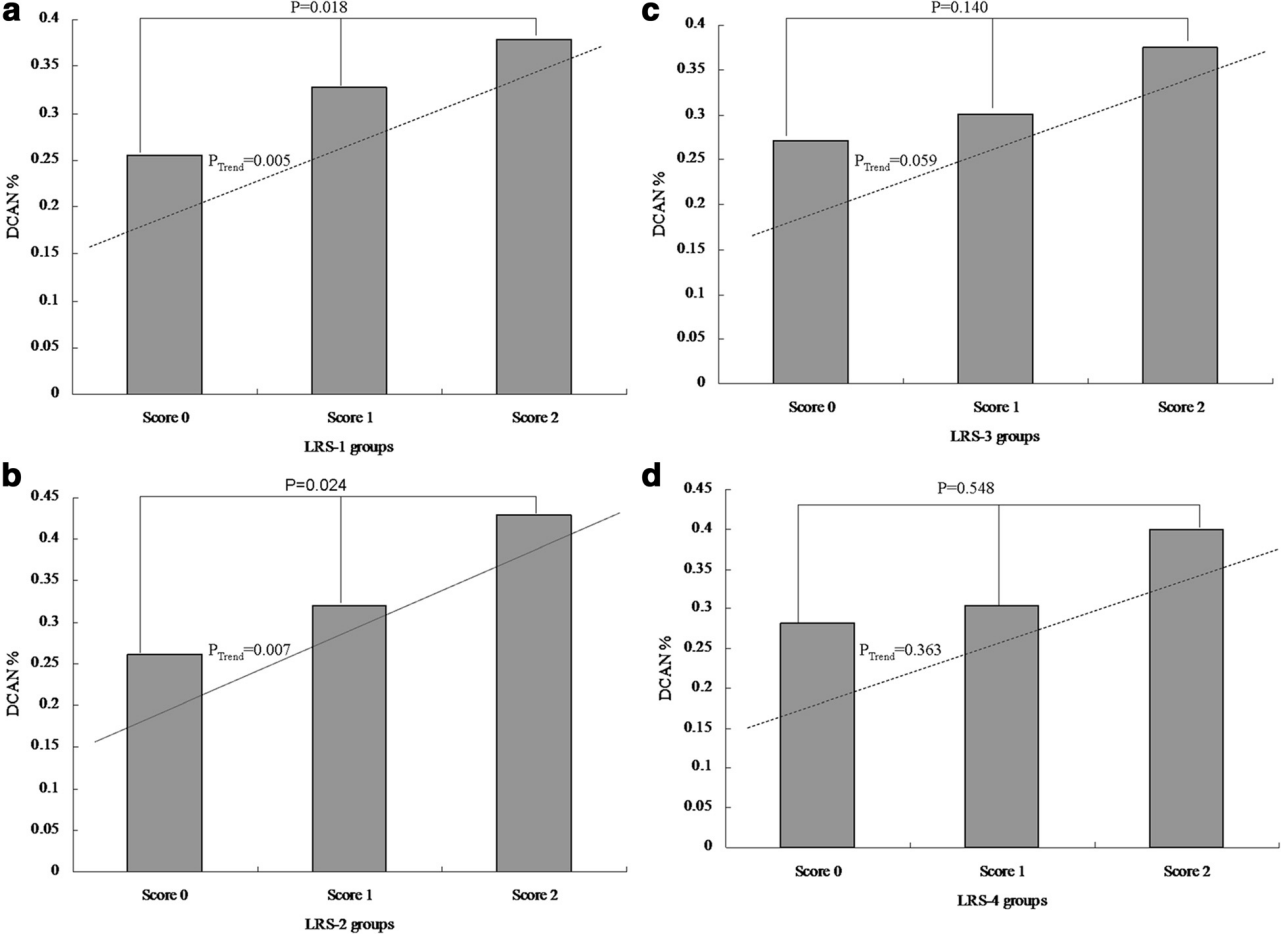
Comparison of prevalence of diabetic cardiovascular autonomic neuropathy (DCAN) according to lipid profile risk score (LRS). **a**: Comparison of DCAN prevalence according to LRS-1. DCAN prevalence was 25.48 %, 32.70 % and 37.84 % in the three groups, respectively. There were significant differences among the three groups (*P* for difference = 0.018 and *P* for a trend =0.005). **b**: Comparison of DCAN prevalence according to LRS-2. DCAN prevalence was 26.18 %, 32.08 % and 42.86 % in the four groups, respectively. There were significant differences among the three groups (*P* for difference = 0.024 and *P* for a trend =0.007). **c**: Comparison of DCAN prevalence according to LRS-3. DCAN prevalence was 27.14 %,30.14 % and 37.50 % in the three groups, respectively. There were no significant differences among the three groups (*P* for difference = 0.140 and *P* for a trend =0.059). **d**: Comparison of DCAN prevalence according to LRS-4. DCAN prevalence was 28.14 %, 30.32 % and 40.00 % in the three groups, respectively. There were no significant differences among the four groups (*P* for difference =0.548 and *P* for a trend =0.363)

There were significant differences among the three LRS-1 groups (25.48 % vs. 32.70 % vs. 37.84 %, *P* for difference = 0.018 and *P* for a trend =0.005, Fig. 5a). Similarly, significant differences were seen among the three LRS-2 groups (26.18 % vs. 32.08 % vs. 42.86 %, *P* for difference = 0.024 and *P* for a trend =0.007, Fig. 5b). In contrast, there were no significant differences among the three groups (27.14 % vs. 30.14 % vs. 37.50 %, *P* for difference = 0.140 and *P* for a trend =0.059, Fig. 5c). Additionally, no significant differences were identified among the three groups (28.14 % vs. 30.32 % vs. 40.00 %, *P* for difference =0.548 and *P* for a trend =0.363, Fig. 5d).

Multiple variables analysis indicated that there was a significant association of severity of LRS-1 or LRS-2 with DCAN (P value =0.058 OR =1.337, 95 % CI: 0.990–1.806 for LRS-1, and P value = 0.079 OR = 1.321, 95 % CI:0.968–1.801 for LRS-2, Table 4). However, there was no significant association of severity of LRS-3 or LRS4 with DCAN (*P <* 0.05 for all).

**Table 4:** The association analysis of severity of lipid profile and diabetic cardiovascular autonomic neuropathy using multiple variable logistic regression analysis

Model	Variable	β	SE	*P* value	OR	95 % CI
Model 1						
	LRS-1	0.291	0.153	0.058	1.337	0.99–1.806
Model 2						
	LRS-2	0.278	0.158	0.079	1.321	0.968–1.801
Model 3						
	LRS-3	0.055	0.155	0.722	1.057	0.78–1.431
Model 4						
	LRS-4	−0.077	0.186	0.680	0.926	0.644–1.333

Note: Model 1: LRS-1 (lipid profile risk score) derived from TC and TG, Model 2: LRS-2 (lipid profile risk score) derived from TG and LDL, Model 3: LRS-3 (lipid profile risk score) derived from TG and HDL, Model 4: LRS-4 (lipid profile risk score) derived from LDL and HDL; BMI-body mass index; all models adjusted for age, gender, smoking, BMI, blood pressure, glucose profile, renal function, medical history

## Discussion

A community-based, cross-sectional study was performed to evaluate the relationship between lipids profile and DCAN in the Chinese population. The prevalence of HTN, MetS, and DCAN in the general populace was similar to that seen in previous studies. As our sample was a good representation of the entire country, our findings might be applied effectively outside the studied areas in China. In this study, we used LRS to combine the information concerning lipids profile to estimate its severity. To the best of our knowledge, this is the first use of LPS to model lipids profile and its severity, in order to explore the extent to which lipids profile is associated with DCAN in the Chinese population. Additionally, short-term HRV was measured non-invasively by a power spectral analysis to evaluate the CA function, due to this test having good reproducibility and greater practicality for application.

Interestingly, our findings signified that TG was significantly and independently associated with DCAN when controlling for confounding factors including age, BMI, glucose profiles, and medical history; however, no association was found between TC, LDH and HDL, and DCAN. In the different indices of lipid profiles, higher levels of the sum of TG and TC, and TG and LDL, were positively related with the severity of DCAN. Another key finding was that the severity of lipids profile was independently and significantly associated with DCAN. There was a tendency toward an increased lipids profile risk score with increasing DCAN prevalence. The MLR model showed that TG, combined with TC and LDL, was associated with this outcome (*P <* 0.1 for LRS-1 and LRS-2).

Diabetic cardiac autonomic neuropathy (DCAN) is a common complication of DM, which is an independent risk factor for cardiovascular and overall mortality, possibly due to increased risk of ventricular arrhythmias and stroke [17, 18]. In T1DM patients, the reported prevalence rates vary between 2.4 % and 36 %[19, 20]. In T2DM patients, the DCAN prevalence is between 30–60 % [13, 21]. In our study, 29.01 % of the enrolled T2DM patients had DCAN, which is consistent with the above published data. However, the previous clinical trials show that DM is not the only risk factor for CAN, because the intensive glucose control can only decrease the incidence of new CAN cases by 53 %[19] and CAN may also occur in newly diagnosed T1DM patients [22]. These results mean that other risk factors are involved in the pathogenesis of CAN, apart from DM.

Dyslipidemia is one such risk factor. Several large-scale clinical studies have indicated that a poor lipids profile is linked with neuropathy development and progression, independent of glycemic control [23–26], and it has recently been identified as a major independent risk factor for the development of neuropathy [27]. In this study, we found that the level of TG is positively related to the prevalence of DCAN in diabetes patients, and can also predict the severity of DCAN when individualized to age, education, and medical and therapy history. In other studies, the relationship between TG and CAN has also been shown. In a Greek population, Voulgari et al. found that CAN occurred in patients with a higher TG level, and that the TG level is positively related to the prevalence of CAN, no matter whether the individual has T1DM or T2DM [28]. The same result was also shown in a population from Finland, in which parasympathetic function detected by a deep-breathing test was used to diagnose CAN [29]. However, some other published data did not find the same connection. Witte et al. found no association between TG and CAN in T1DM patients, but that HDL might have a weak negative relationship with CAN [30]. However, in our study, no correlation between HDL and CAN was found.

Although the relationship between dyslipidemia and neuropathy has been confirmed in clinical trials, the underlying mechanisms by which dyslipidemia damages the neural system are not totally clear. Of these, oxidative stress, as induced by dyslipidemia, is possibly the most important. In rats, a high-fat diet alone can increase oxidative stress, and they develop sensory and motor nerve conduction velocity deficits before the occurrence of impaired glucose tolerance [27]. Enhanced oxidative stress can contribute to the pathology of neural dysfunction in diabetes and has been proposed as a mechanism that contributes to the pathogenesis of neuropathy [31, 32]. A recent study showed the activity of paraoxonase-1 (PON-1),which can reduce hydroperoxides and hydroxyl radicals and is treated as a peroxidase-like enzyme. It decreased in high-fat diet-fed mice, while the activities of MDA, NO levels, SOD, and GPx were enhanced following the increased oxidative stress when they were fed with such a diet [33]. Another mechanism may be related to inflammation TNF-a and TGF-β, two important pro-inflammatory cytokines, were higher in high-fat diet mice than in vehicle-treated mice [33], but whether anti-inflammation chemicals can reduce the progress of neuropathy in diabetic or/and dyslipidemia patients is not yet known.

**S**everal potential limitations of this study should be addressed. First, this is a cross-sectional study exploring the associations among variables, so that we are unable to confirm a direct causal relationship. Additionally, it is important to mention that our study was performed on Chinese individuals, and there is alack of evidence concerning application to other ethnicities. Finally, our findings may be less applicable to younger or elderly populations, due to all subjects being aged between 30 and 80 years.

## Conclusion

Our findings offer evidence that TG and the severity of lipids profile are significantly and independently associated with DCAN, respectively; and that DCAN prevalence was frequent in higher lipids profiles risk scores. These findings indicate that the lipids profile might influence the development of DCAN, providing insight into biological functions.
